# SNP analyses and acoustic tagging reveal multiple origins and widespread dispersal of invasive brown trout in the Falkland Islands

**DOI:** 10.1111/eva.13274

**Published:** 2021-07-16

**Authors:** Jessica F. Minett, Carlos Garcia de Leaniz, Halina Sobolewska, Paul Brickle, Glenn T. Crossin, Sofia Consuegra

**Affiliations:** ^1^ Department of Biosciences Centre for Sustainable Aquatic Research Swansea University Swansea UK; ^2^ South Atlantic Environmental Research Institute (SAERI) Stanley Falkland Islands; ^3^ Noahgene Ltd. Alloa UK; ^4^ School of Biological Science (Zoology) University of Aberdeen Aberdeen UK; ^5^ Department of Biology Dalhousie University Halifax NS Canada

**Keywords:** acoustic telemetry, connectivity, gene flow, genetic diversity, invasive species, population origin, *Salmo trutta*, single nucleotide polymorphisms

## Abstract

Biological invasions are important causes of biodiversity loss, particularly in remote islands. Brown trout (*Salmo trutta*) have been widely introduced throughout the Southern Hemisphere, impacting endangered native fauna, particularly galaxiid fishes, through predation and competition. However, due to their importance for sport fishing and aquaculture farming, attempts to curtail the impacts of invasive salmonids have generally been met with limited support and the best prospects for protecting native galaxiids is to predict where and how salmonids might disperse. We analysed 266 invasive brown trout from 14 rivers and ponds across the Falkland Islands as well as 32 trout from three potential source populations, using a panel of 592 single nucleotide polymorphisms (SNPs) and acoustic tagging, to ascertain their origins and current patterns of dispersal. We identified four genetically distinct clusters with high levels of genetic diversity and low admixture, likely reflecting the different origins of the invasive brown trout populations. Our analysis suggests that many trout populations in the Falklands may have originated from one of the donor populations analysed (River Wey). The highest genetic diversity was observed in the rivers with the greatest number of introductions and diverse origins, while the lowest diversity corresponded to a location without documented introductions, likely colonized by natural dispersal. High levels of gene flow indicated widespread migration of brown trout across the Falkland Islands, likely aided by anadromous dispersal. This is supported by data from acoustically tagged fish, three of which were detected frequently moving between two rivers ~26 km apart. Our results suggest that, without containment measures, brown trout may invade the last remaining refuges for the native endangered *Aplochiton* spp. We provide new insights into the origin and dispersal of invasive brown trout in the Falkland Islands that can pave the way for a targeted approach to limit their impact on native fish fauna.

## 1 INTRODUCTION

The spread of invasive species can occur via accidental introduction, deliberate release and/or natural processes. Controlling biological invasions is increasingly important because they impact native species and communities leading to loss of biodiversity and ecosystem functionality (Doherty et al., 2016; Mills et al., 2003; Mollot et al., 2017), particularly in remote islands with low native diversity (Moser et al., 2018). However, control measures can face social opposition, for example if the costs are high (Sheremet et al., 2017) or the introduced species has acquired socio‐cultural importance (Lohr & Lepczyk, 2014; Roberts et al., 2018). Thus, in some cases, managing the damage caused by invaders can be the best option (Hanley & Roberts, 2019). Management of invasive species requires an understanding of propagule pressure (introduction effort), number of different origins (Du et al., 2021) and pathways and patterns of dispersal (Resh et al., 2018; Sakai et al., 2001). However, unless introductions are deliberate and thorough records are kept, the number and routes of introductions are generally unknown. Molecular techniques, such as microsatellites and single nucleotide polymorphisms (SNPs), can be used to assess the evolution and dispersal of invasive species and design‐targeted plans of containment or eradication (Le Roux & Wieczorek, 2009; Resh et al., 2021).

Brown trout (*Salmo trutta*) is native to Europe, Western Asia and Northern Africa; however, since 1864, it has been widely introduced outside of their native range and is currently found on all continents except Antarctica (MacCrimmon & Marshall, 1968). Such introductions have resulted in extensive ecological damage making brown trout one of the 100 world's worst invasive species (Lowe et al., 2000). Invasive brown trout have had strong negative impacts on native fishes in New Zealand (McDowall, 2006; McIntosh et al., 2000, 2010), Chile (Habit et al., 2010; Penaluna et al., 2009), North America (Budy & Gaeta, 2018; McHugh & Budy, 2006) and Japan (Kitano, 2004; Morita, 2018), causing severe decreases in native biodiversity and loss of ecosystem function through predation, competition and habitat modification (Macchi et al., 2007; Penaluna et al., 2009).

Brown trout from Great Britain (approximately 83,000) and Chile (approximately 30,000‐ with a potential bridgehead effect (Bertelsmeier et al., 2018)) were introduced to the Falkland Islands nearly 80 years ago, over an 18‐year period between 1944 and 1962, although much of the information regarding introduction sites and stocks have been lost. Chilean stocks from Lautaro hatchery were primarily sourced from Germany (Basulto, 2003; Faundez et al., 1997), whereas trout from Great Britain originated from three sources: the Surrey trout farm, Pentlands (Scotland) and the Middleton hatchery in Lancashire (Arrowsmith & Pentelow, 1965; Stewart, 1973, 1980), and included anadromous trout (Minett et al., 2021). The exact sources of the Pentlands stock are unknown but believed to originated from Cobbinshaw Loch or Loch Leven (Minett et al., 2021).

Since their introduction, brown trout have widely spread throughout East and West Falklands (Fowler, 2013; McDowall et al., 2001; Minett et al., 2021). Their spread has been facilitated by marine dispersal, as in other places (Jonsson, 1985; Nevoux et al., 2019), with anadromous brown trout (sea trout) having been documented in the Falklands since 1956 (Salmon & Trout Association, 2012). Additionally, brown trout have been moved intentionally among various locations (McDowall, 2001). The native fish community, mainly zebra trout (*Aplochiton zebra* and *A*. *taeniatus*) and the Falklands minnow (*Galaxias maculatus*), has been severely impacted by brown trout (McDowall et al., 2001), and zebra trout are currently regarded as seriously threatened and protected by law (Falkland Islands Government, 1999; Ross, 2009). However, brown trout can be difficult and costly to eradicate once established (Bosch et al., 2019; Healy et al., 2020) and have become an important source of income through angling tourism in the Falklands (Ross, 2009). Therefore, to maintain a balance between trout fishing and the protection of native galaxiids, targeted management plans should be implemented to limit trout dispersal and prevent further invasion. These need geographical information (e.g. concave and complex coastlines seem to favour brown trout invasion (Labonne et al., 2013)), as well as information on dispersal routes and population connectivity. We analysed the movement and genetic status of brown trout populations across the Falkland Islands to provide information that can be used for future management plans aimed at preventing further dispersal of the species. For this, we used a panel of single nucleotide polymorphisms (SNPs) and acoustic telemetry, to assess population structuring, potential origins of the current brown trout populations and levels of migration and gene flow among them, to establish their patterns of dispersal.

## 2 METHODS

### 2.1 Sampling

**FIGURE 1:**
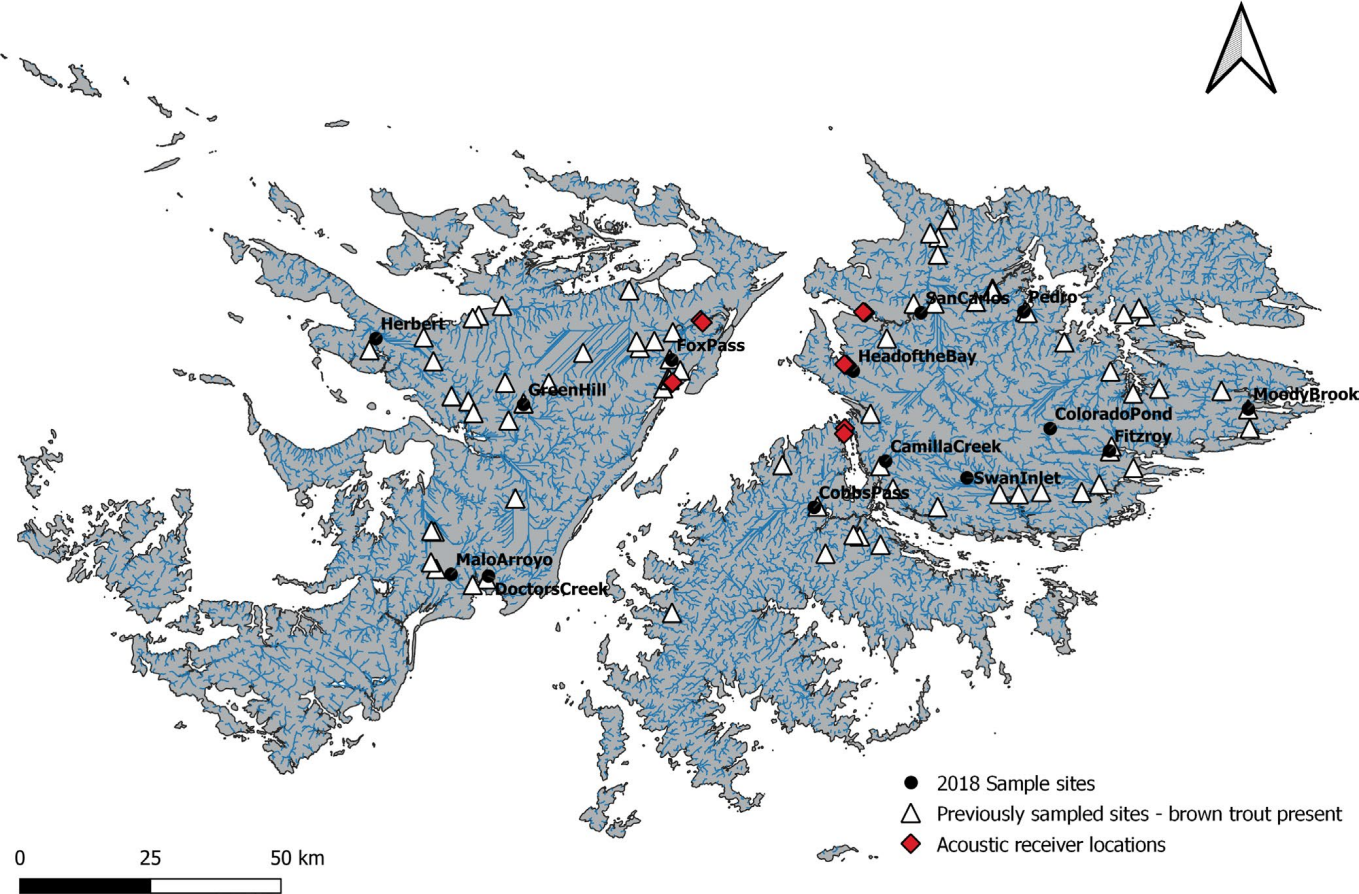
Sampling locations for this study in the Falkland Islands (black circles) and sites where brown trout had been previously detected (white triangle) from McDowall et al. (2001), Ross (2009) and Fowler (2013). Sites where acoustic receiver deployed (red diamonds)

A total of 290 brown trout were non‐lethally sampled from 14 rivers and ponds across the Falkland Islands, nine on East Falkland and five on West Falkland (Figure 1; Table 1), during two field seasons (April–May 2018 [autumn] and September–October 2018 [spring]). Sampling locations were chosen based on brown trout presence information from previous work conducted by McDowall et al. (2001), Ross (2009) and Fowler (2013). Fish were captured using either seine netting, angling or backpack electrofishing (Model: Smith‐Root LR‐24, 160–280 V and 50 Hz). Adipose fin clips and scale samples were obtained from all fish >50 mm, and weight (g) and fork length (mm) were recorded. Fish were then returned alive to their location of capture. Adipose fin clips were stored in 90% ethanol at −20℃ for subsequent genetic analysis.

**TABLE 1:** Details of sampling sites in the Falkland Islands and likely origin of the introduced stocks

River	No. brown trout	No. samples analysed	Sampling method	Island	Introduction stock
Camilla Creek*	25	12	EF	East Falkland	STF/P
Cobbs Pass	22	22	SN	East Falkland	NA
Colorado Pond	25	25	SN & A	East Falkland	NA
Doctors Creek*	24	24	EF	West Falkland	STF/P
Fitzroy*	16	16	EF	East Falkland	STF/P
Fox Pass	17	16	EF	West Falkland	NA
Green Hill*	23	21 (23)	EF	West Falkland	STF/P/MH
Head of the Bay*	25	25	EF	East Falkland	STF/P
Herbert	14	13	EF	West Falkland	NA
Malo Arroyo	9	9	EF	West Falkland	NA
Moody Brook*	11	10	EF	East Falkland	G
Pedro	25	25	EF	East Falkland	NA
San Carlos*	29	14	A	East Falkland	STF/P/MH
Swan Inlet*	25	25	EF & A	East Falkland	STF/P
River Lune	2	(0)		UK	MH
River Wey	12	(12)		UK	STF
Loch Leven	14	(12)		UK	P
Howietoun	6	(6)		UK	P

Note

Number in brackets corresponds to number of samples for Falklands‐GB comparisons when number of samples differed from Falklands‐only analysis. Seine netting (SN), angling (A) and electrofishing (EF). Surrey trout farm (STF), Pentlands (P), German origin from Chile (G) and Middleton Hatchery (MH).

* Indicates introduction sites.

### 2.2 DNA extraction, SNP calling and filtering

DNA from adipose fins was extracted using Qiagen DNeasy 96 plate tissue kits (Qiagen) following the manufacturer's protocol. Concentration and QC parameters were determined using a NanoDrop 8000 spectrophotometer (Thermo Fisher Scientific), and samples normalized to 50 ng/µl. SNP array analysis was completed by Neogen Europe, using a custom design 24k *Salmo salar*/*Oncorhynchus mykiss* Illumina Infinium beadchip array, under permission of Hendrix Genetics (Hendrix Genetics BV.). Data analysis was completed by Noahgene Ltd. Raw data were imported into the software package Genome Studio 2.0.4 (Illumina Inc.) for cluster analysis and SNP calling. SNP calls were exported as a Genome Studio FR.txt file and imported into SNP Variation Suite 8.9.0 (Golden Helix Inc.). QC parameter threshold call rate >0.85 and MAF >0.025 were applied and filtered SNP genotypes (*N* = 14, 195) exported to Microsoft Excel for downstream analysis. Invariant loci (*N* = 12, 233), SNPs with more than 3% missing data overall and any individuals with more than 10% missing data were removed.

### 2.3 Genetic differentiation, isolation by distance and effective population size

Heterozygosity (H_o_), gene diversity (H_s_) and F_IS_ were calculated for each sampling site using the *basic*.*stats* function (Goudet, 2005; Goudet & Jombart, 2020). Deviations from Hardy–Weinberg equilibrium were estimated using the *hw*.*test* (Guo & Thompson, 1992) function from the *adegenet* package. Genetic differentiation between rivers and genetic clusters was calculated using the *hierfstat* 0.5–8 package. Weir and Cockerham pairwise F_ST_ values were calculated using the *pairwise*.*WCfst* function, and 97.5% confidence intervals were obtained by bootstrapping using the *boot*.*ppfst* function (1000 permutations). Overall F_ST_ estimates were calculated using the *betas* function (Weir & Cockerham, 1984). Nei's distance between populations (Nei, 1987) was calculated using the *genet*.*dist* function and used to produce a dendrogram of the population relationships. We estimated effective population size (N_e_) using the linkage disequilibrium method implemented in *NeEstimator* v2 (Do et al., 2014; Hill, 1981).

To examine the extent of isolation by distance (IBD), a Mantel test between genetic distance (pairwise Weir and Cockerham F_ST_ values) and geographic distance matrix was conducted using 999 randomisations in the *ade4* package in R (Mantel, 1967; Thioulouse et al., 2018). We used two measures of geographic distance: pairwise distance between river mouths around the coast (to reflect marine dispersal), and shortest Euclidean distances between sampling sites (to reflect potential human‐mediated translocation of fish). River mouth distances around the coast were calculated using *rgdal* (Bivand et al., 2019), *sp* (Bivand et al., 2013; Pebesma & Bivand, 2005), *raster* (Hijmans, 2020) and *gdistance* (van Etten, 2017) packages using a purpose‐built function. Euclidean distances were calculated in QGIS v3.10.14 (QGIS Development Team, 2020). IBD was calculated for all sampling sites across the Falklands, as well as for East and West Falklands separately. Analyses were carried out using R 3.5.3 (R Core Team, 2019).

### 2.4 Genetic cluster identification, admixture and gene flow

Genetic clusters of related individuals were identified through analysis of SNP genotypes using Discriminant Analysis of Principal Components (DAPC) using the *adegenet* 2.1.3 R package (Jombart, 2008; Jombart & Ahmed, 2011). The optimal number of genetically distinct clusters was determined by K‐means cluster analysis based on the lowest associated Bayesian information criterion (BIC) value, with a maximum K of 14 (the total number of sites sampled in the Falklands), using the *find*.*clusters* function. To examine the genetic structure and describe diversity between clusters, we preformed DAPC using the *dapc* function and the clusters defined by K‐means. The number of principal components retained in DAPC was determined based on their alpha‐scores using the *optim*.*a*.*score* function, resulting in the retention of 5 principal components (Jombart et al., 2010). The level of admixture was assessed through individual assignment to different clusters, assuming that an individual was admixed if it had less than 90% probability of belonging to a single cluster (Noble et al., 2010).

Admixture between clusters was also examined using the *snapclust* function in the *adegenet* R package. Using the clusters defined by K‐means, we simulated F1 and F2 backcrosses between pairs of clusters and *snapclust* was run to reassign individuals to one of six possible classes: parental 1, parental 2, F1 hybrid, F2 or backcross with either parental population.

We calculated directional migration rates as a proxy for gene flow between sampling sites using the *divMigrate* function in the *diveRsity* v1.9.90 R package using genetic diversity and differentiation statistics (Keenan et al., 2013; Sundqvist et al., 2016).

### 2.5 Origin of brown trout introduced into the Falkland Islands

We reconstructed the stocking history of brown trout in the Falkland Islands during 1948–1962 and identified the putative sources from the literature and historical records (Minett et al., 2021). We also obtained fresh or archived tissue samples for genetic analysis from three of the putative sources in Great Britain (Table 1), the River Wey for the Surrey trout farm (*N* = 12) and Howietoun hatchery (*N* = 6) and Loch Leven (*N* = 14) representing Pentlands. We were not able to obtain samples from Cobbinshaw Loch (it was not a natural brown trout population and trout are no longer stocked in the loch) or Germany (the original stock is no longer cultured). DNA extraction and genotyping were carried out as above except for 10 samples obtained from Loch Leven whose DNA had already been extracted. SNP data from putative origins and the Falklands’ samples were combined into a single database and analysed as above to examine genetic clustering and differentiation, using a maximum K of 17.

### 2.6 Acoustic tracking

To gain additional insight into brown trout movements and coastal dispersal around the Falkland Islands, we captured (by angling) and tagged 25 sea trout with size range between 175 and 545 mm from San Carlos River. San Carlos was chosen because it was accessible and surrounded by East and West Falkland rivers with brown trout presence, allowing us to detect movement between the two islands. All fish were tagged with 9 mm ID‐2LP9 acoustic transmitters (Thelma Biotel) inserted in the abdominal cavity, following standard surgical tagging procedures (Lacroix et al., 2005). Tags were programmed to transmit every 180s for approximately 2.5 years and had an acoustic range of ~450 m and a transmitter failure rate reported by manufacturers <2% (Newton et al., 2016). Ten acoustic receivers (VR2W; Vemco Ltd) were deployed in five rivers (two receivers per river; Figure 1) configured to record directional movements for a maximum of 23 months, although two receivers were retrieved after 11 months. To assess movement within and between islands, acoustic receivers were deployed in three sites on East Falkland and two sites on West Falkland, no range testing was conducted.

## 3 RESULTS

### 3.1 Genomic data

We successfully genotyped 265 fish from 14 sites (Table 1) and 32 fish from three putative origins. After removing invariant SNPs and samples with missing data, 477 SNPs were available for the genetic analysis of 257 trout in the Falkland Islands and 592 SNPs for comparisons with three of the putative origins (*N* = 289 trout). The relatively low number of variable SNPs is likely the result of using an array designed for other salmonid species, possibly combined with the history of the introduced populations, that originated from limited number of stocks of hatchery/farm origin, as reflected in the current population structuring. Low intra‐population genetic diversity and high structuring had been also observed in the Falklands brown trout analysed using microsatellites (Monzón‐Argüello, Consuegra, et al., 2014). Eight SNPs (Ax‐87899852, AX‐87986668, AX‐880117788, AX‐88166365, omy19_28513692, omy22_31997564, omy22_39402264, omy_28375016) deviated significantly from Hardy–Weinberg equilibrium in four or more sites in the Falkland Islands, but were retained as their exclusion did not change the genetic clustering of individuals (Figure S1a). For Falklands/GB comparisons, nine SNPs (the same as above in addition to AX‐88095436) deviated significantly from Hardy–Weinberg equilibrium, but were also retained as their exclusion did not affect clustering (Figure S1b).

### 3.2 Genetic differentiation, isolation by distance and effective population size

**FIGURE 2:**
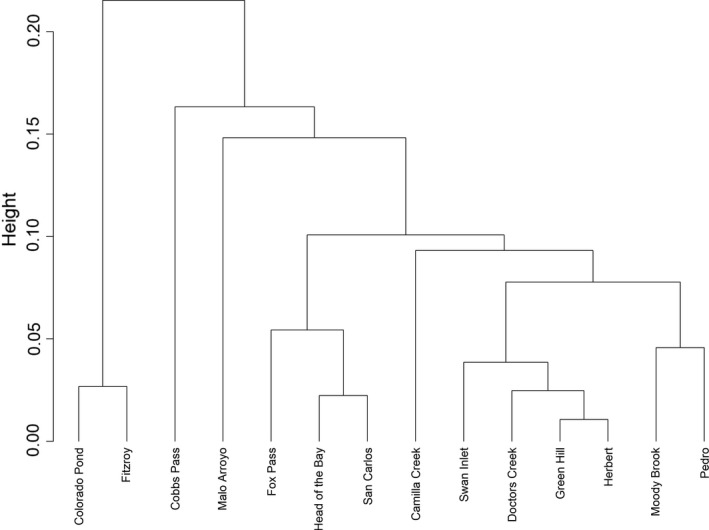
Cluster dendrogram of Falkland Islands sites, based on Nei's distance

The inbreeding coefficient (F_IS_) was negative for all sampling sites, indicating a small excess of heterozygotes (Table 2). Overall F_ST_ for all Falklands sites was 0.09. The smallest pairwise genetic distance (F_ST_ = 0.011) was observed between Green Hill and Herbert, in contrast the largest pairwise genetic distance (F_ST_ = 0.215) observed between Cobbs Pass and Colorado Pond (Figure 2 and Table S1).

**TABLE 2:** Estimates of genetic diversity (observed heterozygosity, H_o_; observed gene diversity, H_s_; F_is_, overall F_ST_) and effective population size (N_e_) calculated according to linkage disequilibrium

Sample site	H_o_	H_s_	F*_Is_*	F_ST_	Sample size	N_e_
Camilla Creek	0.110	0.105	−0.051	0.109	12	inf (inf‐inf)
Cobbs Pass	0.106	0.095	−0.126	0.254	22	20.0 (10.3–55.9)
Colorado Pond	0.095	0.088	−0.080	0.195	25	45.6 (27.4–105.3)
Doctors Creek	0.125	0.116	−0.078	0.009	24	489.0 (167.4‐inf)
Fitzroy	0.115	0.104	−0.106	0.116	16	221.5 (47.2‐inf)
Fox pass	0.121	0.109	−0.116	0.073	16	41.1 (10.6‐inf)
Green Hill	0.116	0.110	−0.055	0.064	21	149.0 (52.8‐inf)
Head of the Bay	0.115	0.105	−0.098	0.108	25	70.6 (36.5–357.7)
Herbert	0.114	0.114	−0.002	0.029	13	inf (112.6‐inf)
Malo Arroyo	0.109	0.104	−0.046	0.114	9	inf (265.9‐inf)
Moody Brook	0.108	0.104	−0.035	0.116	10	11.5 (3.0–965.3)
Pedro	0.120	0.114	−0.050	0.028	25	130.7 (69.4–698.0)
San Carlos	0.129	0.121	−0.061	−0.032	14	16.3 (7.1–80.5)
Swan Inlet	0.112	0.109	−0.033	0.077	25	111.9 (60.3–521.4)

Estimates of N_e_ based on linkage disequilibrium ranged from 11.5 (95% CI = 3–965) at Moody Brook to 489 (95% CI = 167‐infinity) at Doctors Creek (Table 2).

No significant isolation by distance was found, using either geographic distance around the coast (*r* = 0.085; *p* = 0.272) or Euclidean distance (*r* = −0.042; *p* = 0.593), for the Falklands together or for West Falkland (coastal distance *r* = −0.084; *p* = 0.601; Euclidean distance *r* = 0.144; *p* = 0.343). However, a significant correlation was observed for rivers in East Falkland between genetic and coastal distance (*r* = 0.391; *p* = 0.004) but not for Euclidean distance (*r* = 0.282; *p* = 0.116), suggesting a role for marine dispersal.

### 3.3 Genetic cluster identification, admixture and gene flow

**FIGURE 3:**
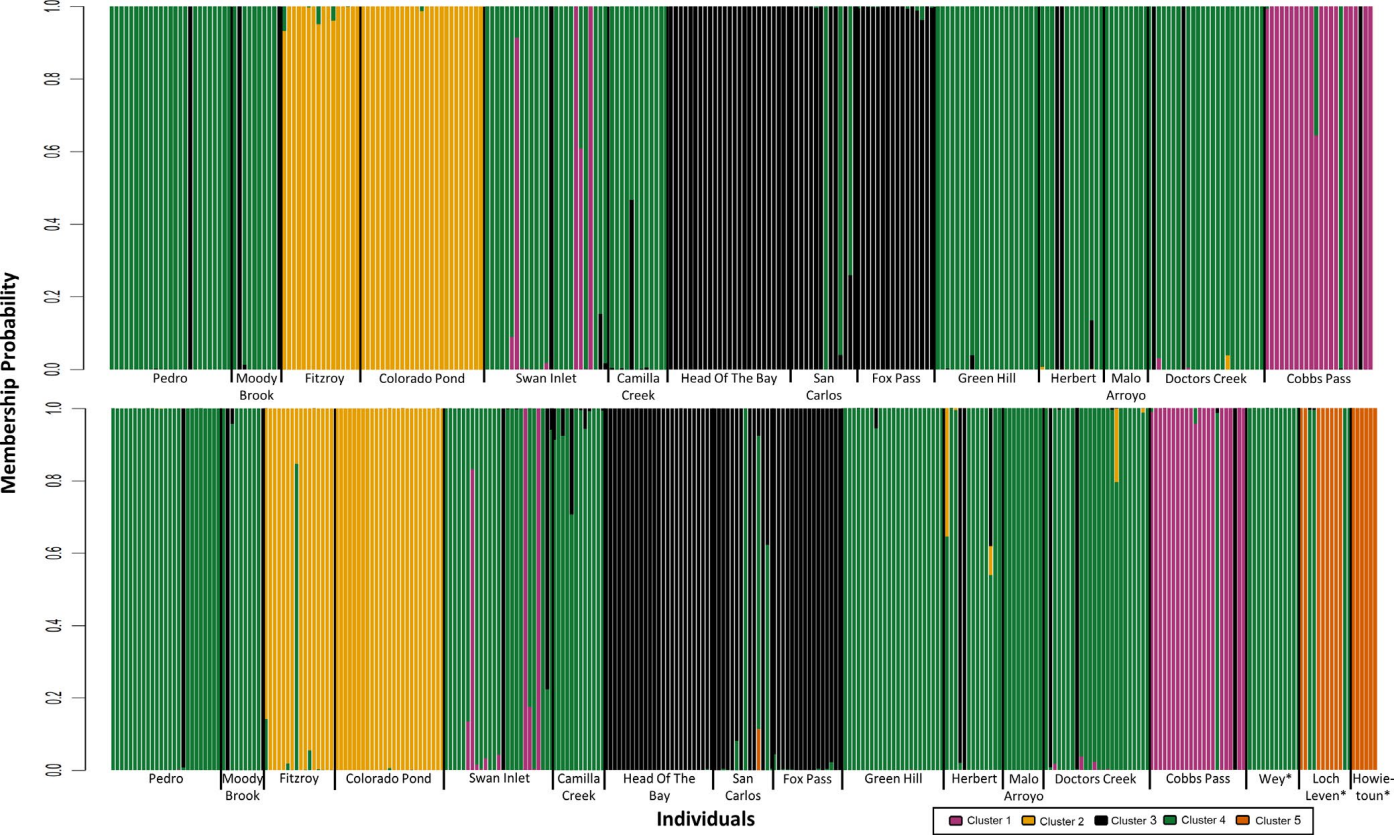
Discriminant Analysis of Principal Components (DAPC) analysis of population structure for (a) Falkland Islands brown trout based on 477 SNPs and *K* = 4 and (b) Falkland Islands and GB brown trout based on 592 SNPs and *K* = 5. Each bar corresponds to an individual, and colours represent genetic clusters. (*) Indicate GB sites

Results of the DAPC analysis support four genetically distinct genetic clusters of brown trout in the Falkland Islands (Figure 3a, *K* = 4 BIC = 1983.383; Figure S2a). Cobbs Pass largely formed its own cluster, cluster 1, which contained 25 individuals including a few from Swan Inlet. One cluster (cluster 2) contained 41 individuals from Colorado Pond and Fitzroy and was well differentiated from the rest of the sampling sites (Table 3). Another cluster (cluster 3) consisted of 61 individuals primarily from Fox Pass, Head of the Bay and San Carlos. The remaining fish formed cluster 4, which contained 130 individuals from all sampling sites except Colorado Pond, Fox Pass and Head of the Bay. All clusters were clearly distinct (Figure S3a). The lowest pairwise distance was observed between cluster 1 and cluster 4 (F_ST_ of 0.081) and the largest between cluster 1 and cluster 2 (F_ST_ of 0.202, Table 3).

**TABLE 3:** Pairwise F_ST_ values for clusters (*K* = 4) of Falkland Islands samples, calculated according to Weir and Cockerham

	Cluster 1	Cluster 2	Cluster 3
Cluster 2	0.202		
Cluster 3	0.122	0.136	
Cluster 4	0.081	0.088	0.046

Only six individuals from five sites displayed evidence of admixture between the distinct genetic backgrounds (clusters), mostly between clusters 3 and 4 and between clusters 1 and 4, with the greatest number of admixed individuals being from Swan Inlet (Figure 3). Admixed individuals included 9% F1 hybrids and 0.6%–28% backcrosses (Figure S4).

Estimates of migration rates were consistent with the DAPC results (Table 4; Figure S5). The greatest inferred gene flow was between Head of the Bay and San Carlos (Nm = 1.00), while Malo Arroyo was the only clearly isolated site (Nm ≤ 0.26).

**TABLE 4:** Relative migration rates (from row to column) between Falkland Islands sites, calculated using Nm

	Camilla Creek	Cobbs Pass	Colorado Pond	Doctors Creek	Fitzroy	Fox Pass	Green Hill	Head of the Bay	Herbert	Malo Arroyo	Moody Brook	Pedro	San Carlos	Swan Inlet
Camilla Creek		0.19	0.12	0.29	0.14	0.27	0.28	0.24	0.38	0.16	0.18	0.34	0.32	0.38
Cobbs Pass	0.22		0.12	0.30	0.14	0.17	0.25	0.20	0.25	0.13	0.14	0.29	0.23	0.50
Colorado Pond	0.16	0.13		0.25	0.81	0.15	0.27	0.17	0.27	0.14	0.16	0.24	0.17	0.21
Doctors Creek	0.29	0.26	0.22		0.26	0.36	0.71	0.38	0.71	0.23	0.29	0.50	0.44	0.58
Fitzroy	0.18	0.15	0.74	0.32		0.19	0.32	0.20	0.32	0.15	0.22	0.30	0.20	0.23
Fox pass	0.32	0.21	0.15	0.40	0.17		0.35	0.46	0.35	0.16	0.25	0.37	0.78	0.37
Green Hill	0.35	0.27	0.21	0.89	0.25	0.33		0.33	0.89	0.25	0.30	0.67	0.49	0.67
Head of the Bay	0.28	0.22	0.16	0.40	0.15	0.56	0.36		0.37	0.14	0.28	0.36	1.00	0.36
Herbert	0.43	0.28	0.23	0.84	0.26	0.36	0.95	0.40		0.22	0.32	0.64	0.50	0.57
Malo Arroyo	0.16	0.14	0.13	0.24	0.14	0.16	0.24	0.16	0.21		0.17	0.26	0.20	0.21
Moody Brook	0.22	0.15	0.17	0.25	0.19	0.22	0.31	0.23	0.28	0.17		0.44	0.26	0.29
Pedro	0.36	0.25	0.20	0.54	0.26	0.31	0.68	0.32	0.62	0.24	0.36		0.43	0.50
San Carlos	0.25	0.23	0.15	0.35	0.17	0.48	0.34	0.49	0.35	0.16	0.25	0.37		0.35
Swan Inlet	0.36	0.45	0.20	0.72	0.25	0.33	0.64	0.36	0.59	0.22	0.28	0.57	0.49	

### 3.4 Genetic assignment to putative populations of origin

The results of the DAPC analysis of samples from both the Falkland Islands and Great Britain indicated the existence of five genetically distinct clusters (Figure 3b and Figures S2b and S3b), the 4 clusters identified in the Falklands‐only analysis and one additional cluster (5), which consisted of individuals from Loch Leven and the Howietoun hatchery (Figure 3). Trout from the River Wey, representative of the Surrey trout farm, were included in cluster 4, suggesting that they could be the origin of the trout populations in Camila Creek, Doctors Creek, Green Hill, Herbert, Malo Arroyo, Moody Brook, Pedro and Swan Inlet. Despite Loch Leven samples forming part of cluster 5, four of the 12 individuals belonged to cluster 4, suggesting Loch Leven as another possible source of these populations. The smallest genetic distance was between clusters 2 and 4 (F_ST_ = 0.047) and the greatest between clusters 1 (Falklands) and 5 (Great Britain; F_ST_ = 0.205, Table 5).

**TABLE 5:** Pairwise F_ST_ values for clusters (*K* = 5) of Falkland Islands‐GB comparisons, calculated according to Weir and Cockerham

	Cluster 1	Cluster 2	Cluster 3	Cluster 4
Cluster 2	0.117			
Cluster 3	0.193	0.131		
Cluster 4	0.079	0.047	0.082	
Cluster5	0.205	0.167	0.185	0.129

### 3.5 Acoustic tracking

We detected movements of 12 fish (mean length 365 mm) in San Carlos (Table S2), three of which also moved to Head of the Bay (separated by 26 km around the coast), confirming the migration between different rivers through marine dispersal. The remaining 13 fish (mean length 367 mm) were not detected on any of the acoustic receivers. Of the three fish that were detected in both San Carlos and Head of the Bay, two fish moved from San Carlos to Head of the Bay and back. A third fish moved between the two sites twice and was detected around Head of the Bay initially for 22 days before being detected in San Carlos 26 days later.

## 4 DISCUSSION

Our analysis revealed the presence of four genetically distinct clusters of invasive brown trout in the Falkland Islands, likely reflecting their different origins, although we cannot discard the effects of founder effects on the structuring. The four clusters had high levels of genetic diversity and low levels of admixture, although high levels of gene flow were detected between rivers within each cluster. We also observed variable effective population sizes (N_e_ ranging from 12 to 489), with high 95% confidence intervals in several cases, potentially due to low sample sizes (Do et al., 2014). These estimates were greater than those estimated by a previous study 10 years ago, for which N_e_ ranged between 16 and 46 (Monzón‐Argüello, Consuegra, et al., 2014). The difference in N_e_ could reflect the expansion of brown trout in the Falklands, potentially aided by marine dispersal as evidenced from our estimates of numbers of migrants, supported by acoustic tagging. However, this comparison must be treated with caution as there were differences in the molecular markers and methods used in both studies (microsatellites and maximum likelihood in 2014, SNPs and linkage disequilibrium method here), as well as in the target populations. Yet, both our current and previous analysis identified strong population structuring, lack of isolation by distance and the presence of anadromous trout (Monzón‐Argüello, Consuegra, et al., 2014).

We identified putative F1 and backcrossed individuals between two genetic clusters (cluster 2 and 4) that could have resulted from marine dispersal, but also from admixture with farmed escapees. One of the rivers in cluster 2 (Fitzroy) is close to the location where sea trout are being farmed in open net cages since 2013, initially with locally captured brood stock from Camilla Creek (cluster 4), and then with ova imported from Howietoun Hatchery UK in 2014 and 2015. Although we found no direct evidence of mixing between Fitzroy and Howietoun fish, escapes from sea pens are not uncommon in salmonid farming and are the main route of introduction of invasive salmonids in the Southern Hemisphere (Arismendi et al., 2009; Consuegra et al., 2011; Monzón‐Argüello et al., 2014). Thus, the presence of farmed fish in close proximity to naturalized populations could have resulted in admixture, as for rainbow trout in Chile (Consuegra et al., 2011; Monzón‐Argüello et al., 2013), potentially increasing dispersal. Alterations in dispersal patterns can be expected by genetic admixture between wild (naturalized in this case) and captive‐bred trout (Saint‐Pé et al., 2018) and could apply to this this case, considering the anadromous nature of the farmed stock.

The highest level of genetic diversity was observed in trout from the rivers San Carlos and Green Hill. These are the sites with the greatest number of documented introductions and the most diverse origins, including three sources from Great Britain (Arrowsmith & Pentelow, 1965; Fowler, 2013; Stewart, 1973). In comparison, lower genetic diversity was observed in Cobbs Pass, where no fish were stocked and where the population likely represents the result of secondary invasions and natural dispersal, as suggested by earlier studies in the Falkland Islands (Monzón‐Argüello, Consuegra, et al., 2014; Monzon‐Arguello et al., 2014) and the Kerguelen Islands, where current genetic diversity largely reflects the pattern of historical introductions (Launey et al., 2010).

Our analysis of some of the donor populations from Great Britain suggests that many trout in the Falklands must have originated from the River Wey (representative of the Surrey trout farm) as reported in the early literature (Arrowsmith & Pentelow, 1965; Stewart, 1973) and, to a lesser extent, possibly from Loch Leven. Although we did not have samples from two other potential sources (Middleton hatchery or Cobbinshaw Loch), our results indicate that trout in the Falklands likely originate from four distinct sources that can be used to trace new colonization events. Migration between sampling sites could have been the result of human‐mediated translocations, as reported by McDowall et al. (2001). However, strong runs of sea trout have been observed since 1956 (Salmon & Trout Association, 2012) indicating the ability of brown trout to colonize new rivers through marine dispersal (Launey et al., 2010; Westley & Fleming, 2011), a fact also supported by our results of acoustically tagged fish, which were detected moving between two rivers ~26 km apart (San Carlos and Head of the Bay). A recent study using environmental DNA (eDNA) has detected brown trout in sites where the species had not previously been recorded, suggesting it is continuing to spread across the Falklands (Minett et al., 2020). Without containment measures in place, there is a risk that brown trout may invade the last remaining refuges for the native endangered *Aplochiton* spp.

The popularity of brown trout as sport fish is common to other countries where it is also highly invasive, such as United States or New Zealand (Jones & Closs, 2018), but awareness of the negative impacts on the native ecosystems is increasing, and plans for containment or eradication are starting to be implemented (Budy & Gaeta, 2018; Saunders et al., 2015). Mechanical removal of trout tends to increase the density of small fish (Meyer et al., 2006; Saunders et al., 2015) and is not an appropriate managing strategy, but they seem to be contained by beaver dams and natural competition at high densities of native fish (Budy & Gaeta, 2018). A combination of molecular markers, eDNA and acoustic tagging, as used here, can be employed to monitor the expansion of brown trout and put measures in place to limit its dispersal, for example through the installation of selective barriers (Jones et al., 2021).

## CONFLICT OF INTEREST

None declared.

## Supporting information

Supplementary Material

## Data Availability

The data that support the findings of this study are openly available in Figshare under https://doi.org/10.6084/m9.figshare.14851911.
