# Supplementary material for: SNP analyses and acoustic tagging reveal multiple origins and widespread dispersal of invasive brown trout in the Falkland Islands
Source: Evol Appl. 2021 Jul 16;14(8):2134–44. doi: 10.1111/eva.13274 (PMC8372121; doi:10.1111/eva.13274)
Supplement: Supplementary file 1 — Supplementary Material [file EVA-14-2134-s001.docx]

**Supplementary Material – SNP analyses and acoustic tagging reveal multiple origins and widespread dispersal of invasive brown trout in the Falkland Islands**

**Table S1.** Pairwise F_ST_ values for Falkland Islands sample sites, calculated according to Weir and Cockerham.

|  | **Camilla Creek** | **Cobbs Pass** | **Colorado Pond** | **Doctors Creek** | **Fitzroy** | **Fox Pass** | **Green Hill** | **Head of the Bay** | **Herbert** | **Malo Arroyo** | **Moody Brook** | **Pedro** | **San Carlos** |
| --- | --- | --- | --- | --- | --- | --- | --- | --- | --- | --- | --- | --- | --- |
| **Cobbs Pass** | 0.133 |  |  |  |  |  |  |  |  |  |  |  |  |
| **Colorado Pond** | 0.172 | 0.215 |  |  |  |  |  |  |  |  |  |  |  |
| **Doctors Creek** | 0.083 | 0.100 | 0.121 |  |  |  |  |  |  |  |  |  |  |
| **Fitzroy** | 0.146 | 0.181 | 0.027 | 0.083 |  |  |  |  |  |  |  |  |  |
| **Fox pass** | 0.074 | 0.143 | 0.170 | 0.065 | 0.127 |  |  |  |  |  |  |  |  |
| **Green Hill** | 0.069 | 0.103 | 0.126 | 0.025 | 0.095 | 0.072 |  |  |  |  |  |  |  |
| **Head of the Bay** | 0.101 | 0.136 | 0.172 | 0.075 | 0.137 | 0.055 | 0.083 |  |  |  |  |  |  |
| **Herbert** | 0.049 | 0.105 | 0.115 | 0.020 | 0.080 | 0.066 | 0.011 | 0.069 |  |  |  |  |  |
| **Malo Arroyo** | 0.122 | 0.168 | 0.177 | 0.089 | 0.146 | 0.130 | 0.088 | 0.149 | 0.093 |  |  |  |  |
| **Moody Brook** | 0.093 | 0.169 | 0.153 | 0.071 | 0.106 | 0.091 | 0.064 | 0.096 | 0.062 | 0.109 |  |  |  |
| **Pedro** | 0.067 | 0.109 | 0.132 | 0.050 | 0.092 | 0.078 | 0.031 | 0.088 | 0.027 | 0.086 | 0.044 |  |  |
| **San Carlos** | 0.063 | 0.110 | 0.158 | 0.052 | 0.122 | 0.024 | 0.045 | 0.023 | 0.042 | 0.103 | 0.073 | 0.054 |  |
| **Swan Inlet** | 0.068 | 0.061 | 0.141 | 0.037 | 0.111 | 0.074 | 0.036 | 0.081 | 0.039 | 0.096 | 0.077 | 0.052 | 0.049 |

**Table S2.** Breakdown of acoustic receiver detection data in the Falkland Islands at three sites.

| Receiver | River | Date range | Transmitter ID | Number of detections | Total number of detections | Latitude | Longitude |
| --- | --- | --- | --- | --- | --- | --- | --- |
| VR2W-132982 | San Carlos | 18/02/19 – 12/11/20 | A69-1303-4439 | 4 | 2567 | -51.5069 | -58.9757 |
|  |  | 20/01/19 – 16/11/19 | A69-1303-4441 | 209 |  |  |  |
|  |  | 20/02/19 – 25/02/19 | A69-1303-4448 | 635 |  |  |  |
|  |  | 28/11/19 – 02/01/20 | A69-1303-4450 | 8 |  |  |  |
|  |  | 29/12/19 – 03/01/20 | A69-1303-4453 | 115 |  |  |  |
|  |  | 24/02/19 – 18/10/20 | A69-1303-4454 | 3 |  |  |  |
|  |  | 20/01/19 – 17/02/19 | A69-1303-4457 | 97 |  |  |  |
|  |  | 20/01/19 – 16/02/19 | A69-1303-4458 | 1488 |  |  |  |
|  |  | 08/03/19 – 12/12/19 | A69-1303-4459 | 6 |  |  |  |
|  |  | 23/01/20 | A69-1303-4460 | 1 |  |  |  |
|  |  | 12/11/19 | A69-1303-4461 | 1 |  |  |  |
| VR2W-132983 | San Carlos | 18/02/19 | A69-1303-4439 | 1 | 986 | -51.5057 | -58.9811 |
|  |  | 20/01/19 – 17/11/19 | A69-1303-4441 | 51 |  |  |  |
|  |  | 21/01/19 – 22/02/19 | A69-1303-4448 | 14 |  |  |  |
|  |  | 28/11/19 – 03/01/20 | A69-1303-4450 | 6 |  |  |  |
|  |  | 29/12/19 – 03/01/20 | A69-1303-4453 | 39 |  |  |  |
|  |  | 27/01/19 – 01/02/19 | A69-1303-4457 | 29 |  |  |  |
|  |  | 20/01/19 – 15/02/19 | A69-1303-4458 | 836 |  |  |  |
|  |  | 01/02/19 – 12/12/19 | A69-1303-4459 | 4 |  |  |  |
|  |  | 05/11/19 – 23/01/20 | A69-1303-4460 | 4 |  |  |  |
|  |  | 12/11/19 | A69-1303-4461 | 1 |  |  |  |
|  |  | 12/12/19 | A69-1303-4466 | 1 |  |  |  |
| VR2W-132983 | Head of the Bay | 10/11/19 | A69-1303-4439 | 2 | 28 | -51.5947 | -59.0370 |
|  |  | 09/11/19 | A69-1303-4454 | 1 |  |  |  |
|  |  | 04/12/19 – 09/11/20 | A69-1303-4460 | 25 |  |  |  |


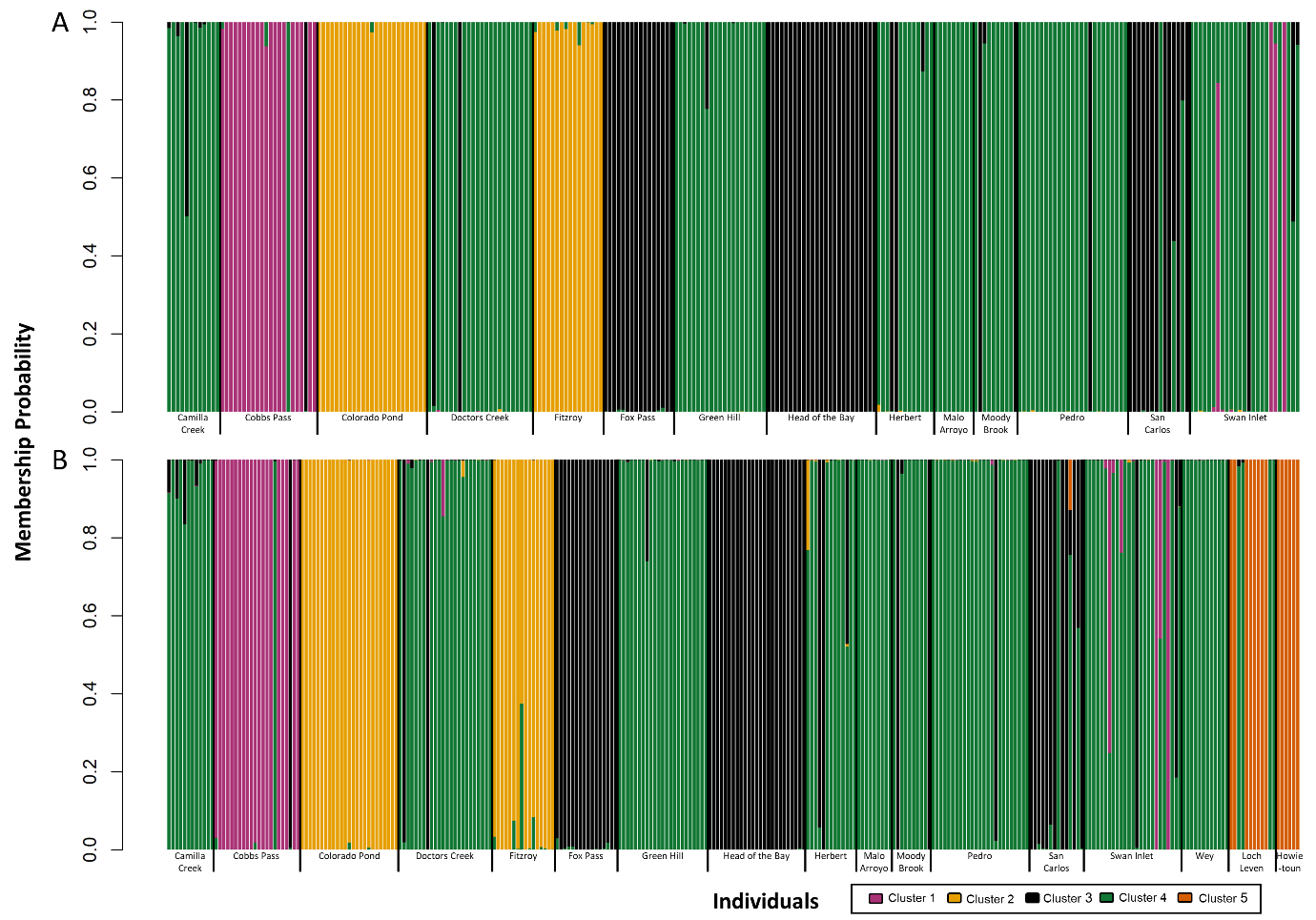


**Figure S1**. Population structure for Falkland Islands (A) and Falklands and UK (B) with SNPs that significantly deviate from Hardy-Weinberg equilibrium removed. A total of eight and nine SNPs were removed from Falkland Islands only and Falklands and Great Britain comparisons, respectively.


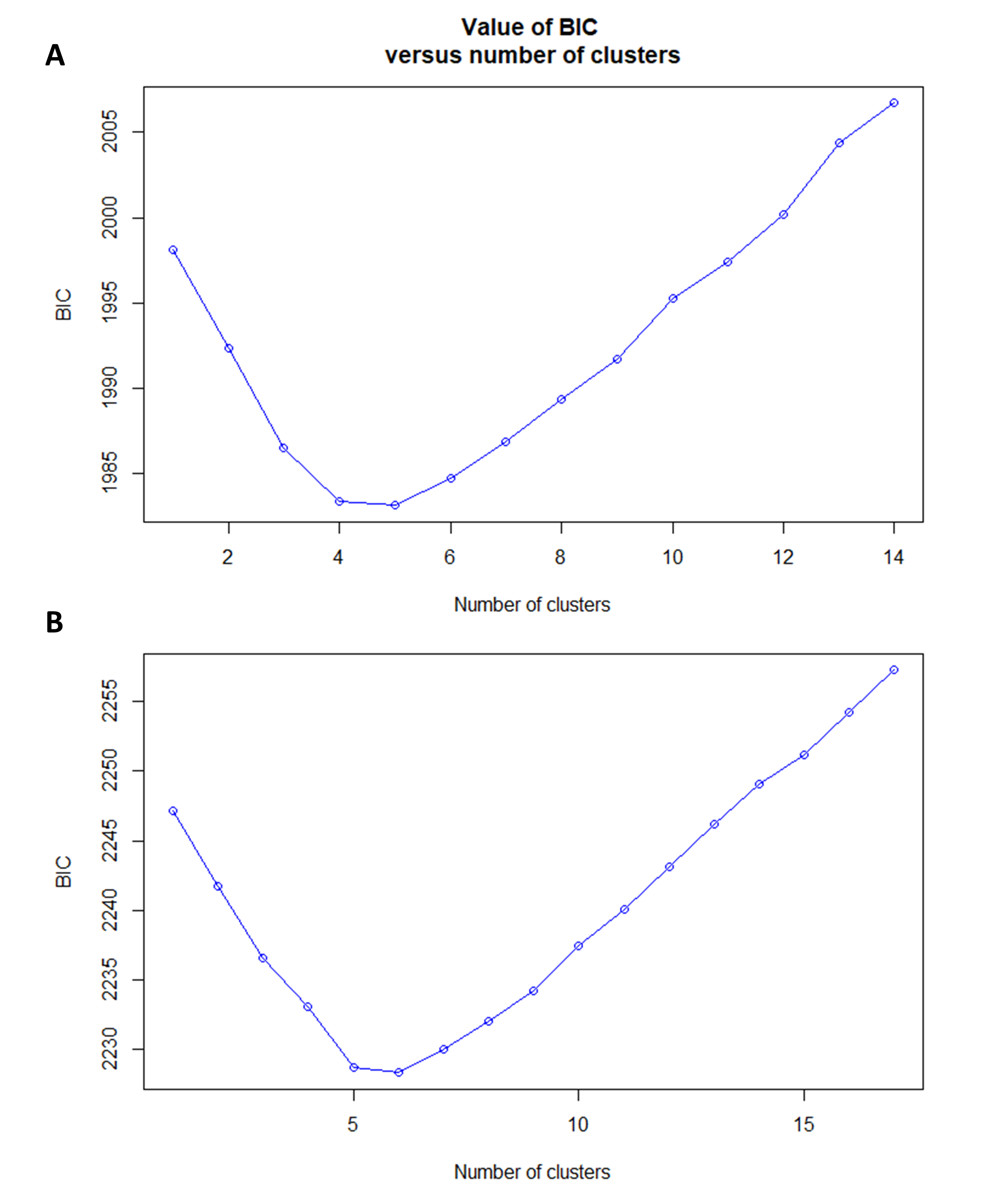
**Figure S2**. Bayesian Information Criterion (BIC) values for the different number of clusters in (A) Falkland Islands only data (optimal K = 4) and (B) Falkland Islands GB Comparisons, (optimal K = 5).


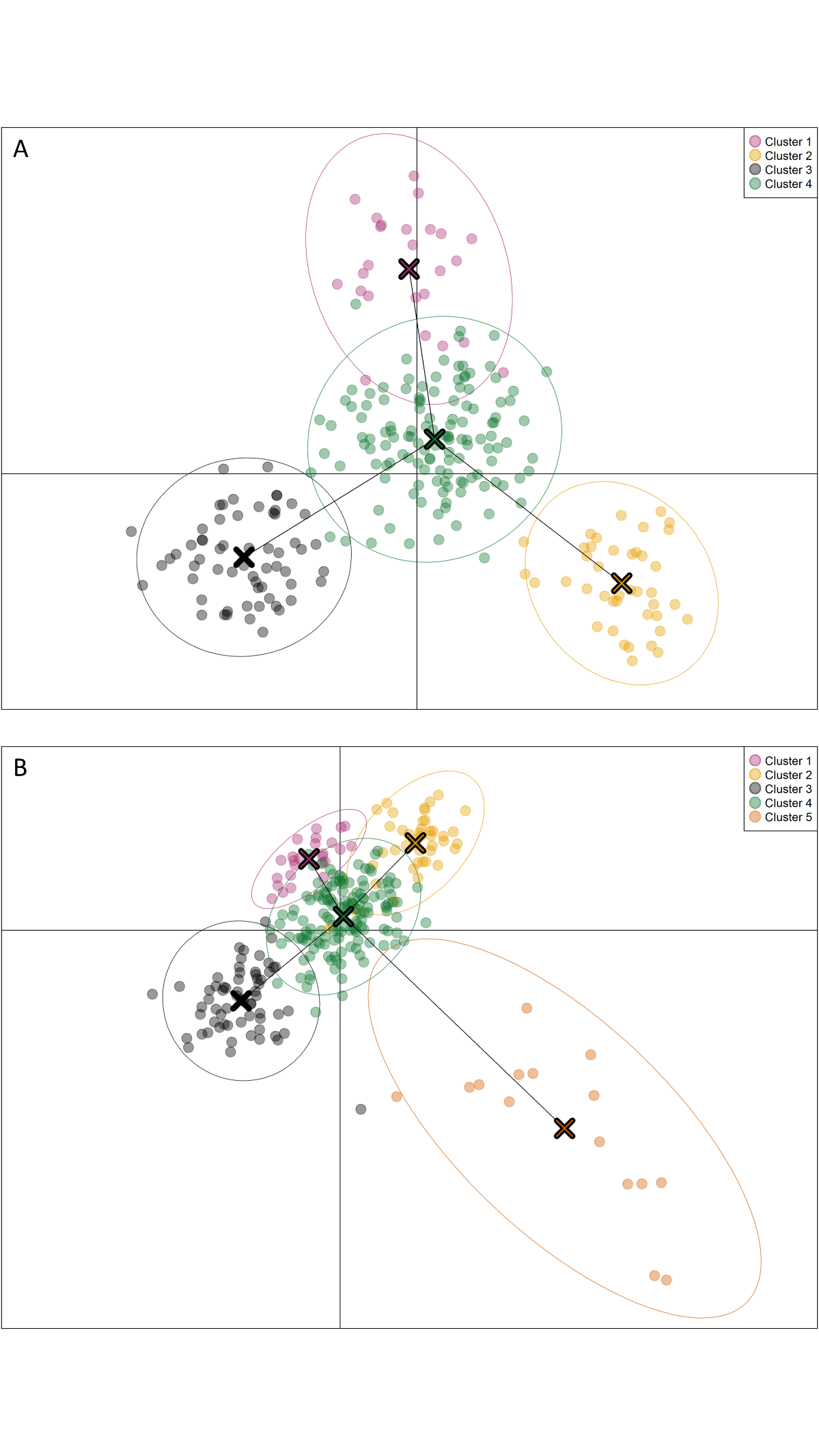


**Figure S3**. Discriminant analysis of principal components (DAPC) for brown trout in (A) the Falkland Islands, based on 477 SNPs and (B) Falkland Islands and Great Britain, based on 592 SNPs. Dots represent individuals.


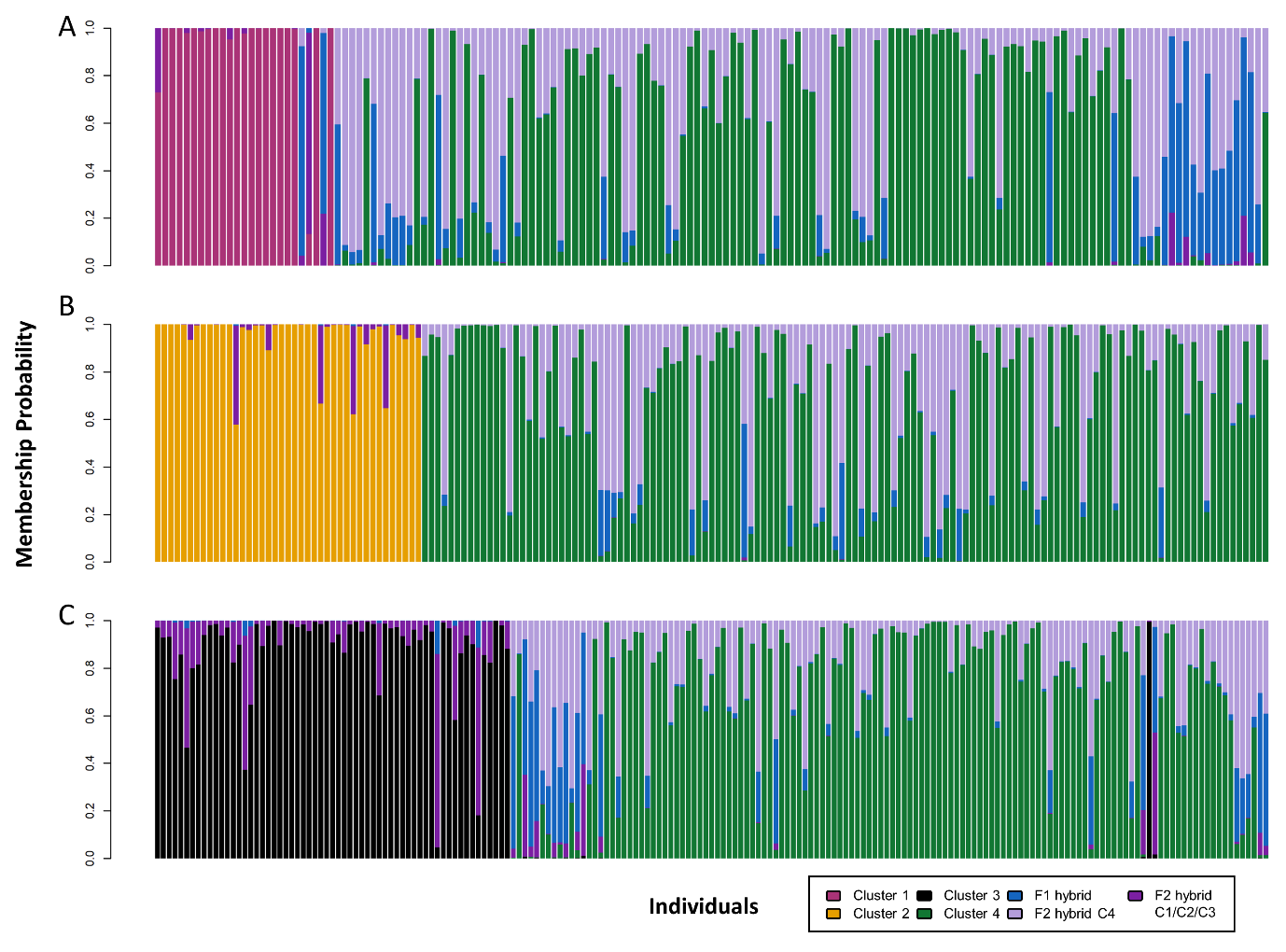


**Figure S4.** Population structure of brown trout in the Falkland Islands when F1 (hybrids between clusters) and F2 (backcrosses with original clusters) are accounted for. Hybridisation analysed between cluster 1 and 4 (A), cluster 2 and 4 (B), and cluster 3 and 4 (C). Bars represent individuals and colours represent cluster and hybrids.


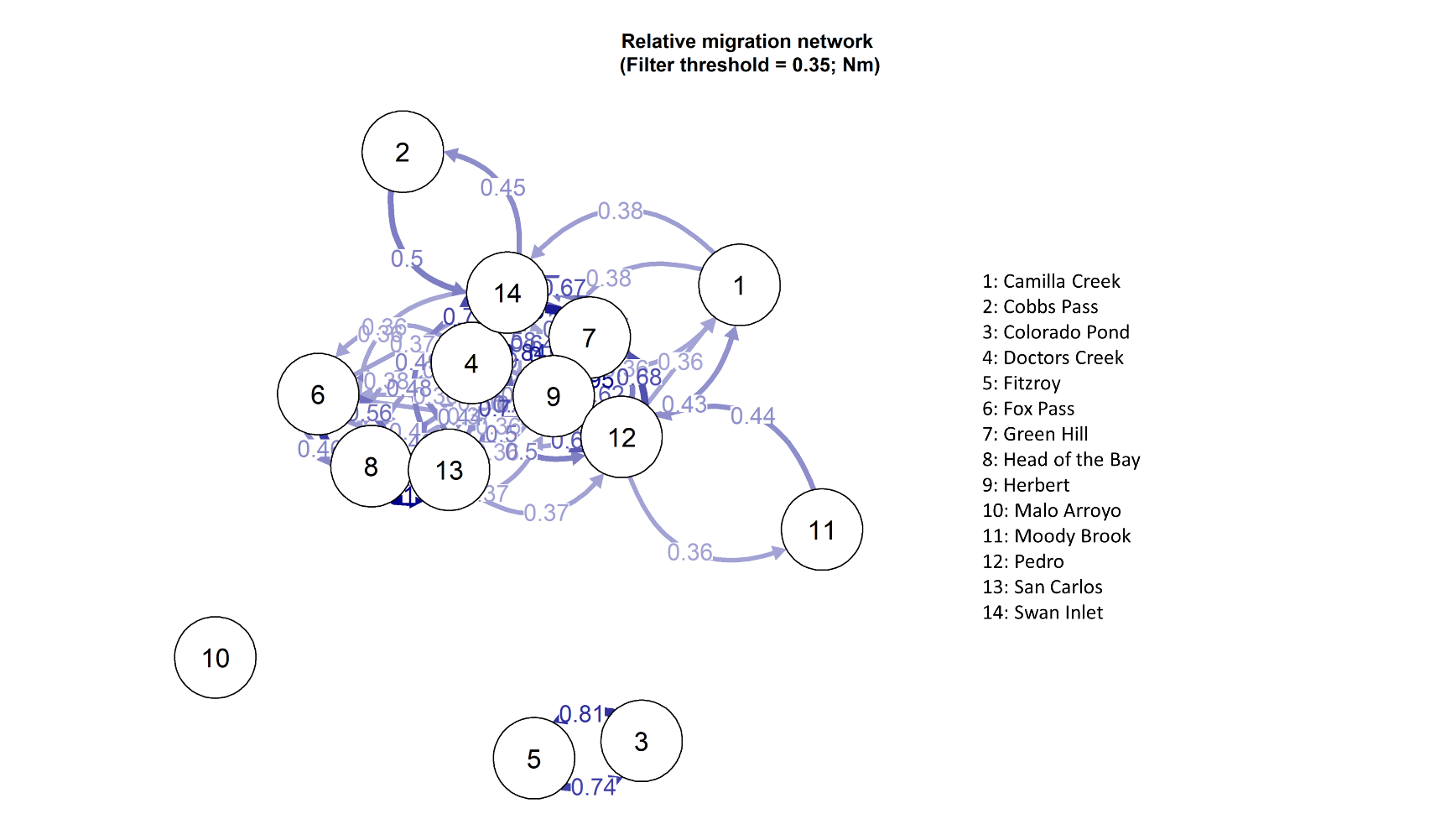


**Figure S5.** Relative migration network among Falkland Islands sampled rivers/lakes, migration rates calculated using Nm, threshold set to 0.35.
